# Comparison of the Presence of Heavy Metal Resistance Genes in *Salmonella enterica* and Their Association with Antibiotic Resistance

**DOI:** 10.3390/microorganisms13122696

**Published:** 2025-11-26

**Authors:** Eric Tang, Ashraf Khan, Steven L. Foley

**Affiliations:** 1 Little Rock Central High School, Little Rock, AR 72202, USA; eric.tang367@outlook.com; 2 Division of Microbiology, FDA National Center for Toxicological Research, Jefferson, AR 72079, USA; ashraf.khan@fda.hhs.gov

**Keywords:** copper resistance, silver resistance, mercury resistance, arsenic resistance, antimicrobial resistance, plasmids, United States

## Abstract

Metals are widely used in animal feed for their growth-stimulating and antimicrobial effects; yet, there is potential concern that their use can promote antimicrobial resistance through co-selection. However, the prevalence of these metal resistance genes in *Salmonella* and their impact on the induction of antimicrobial resistance remain unclear. To aid in this understanding, this study investigated of the prevalence of heavy metal resistance genes (HMRGs) and their comparison with antimicrobial resistance genes (ARGs) in *Salmonella enterica* strains isolated from various sources, across different locations and time periods. Data on stress and AMR genotypes, serovar, source, location, and collection date were retrieved from the NCBI Pathogen Detection Isolate Browser. Isolates from the United States with complete metadata were analyzed using Microsoft Excel and PANDAS (Python Data Analysis Library). Chi-square tests were conducted to assess differences in ARG presence between HMRG-positive and HMRG-negative isolates. Additionally, the co-localization of HMRGs and ARGs on plasmids was examined, and plasmid incompatibility types were assessed. The results show that HMRG prevalence varied significantly across serovars and sources. Certain ARGs occurred at significantly higher frequencies in isolates harboring HMRGs. Co-localization of HMRGs and ARGs on plasmids was frequently observed, although no specific plasmid incompatibility group was uniquely associated with this co-localization. These findings highlight a potential link between metal resistance and antibiotic resistance in *S. enterica*, reinforcing concerns about the use of heavy metals in agriculture. The results provide important insights for risk assessment and inform strategies aimed at mitigating AMR as a global public health threat.

## 1. Introduction

Antibiotics have traditionally been used to treat animal diseases and as growth promoters. However, the rise in antimicrobial resistance (AMR), particularly multidrug resistance, has become a global health issue that threatens the efficacy of antibiotics and modern medicine [1]. Groups including the World Health Organization (WHO), regional governmental unions, and individual countries have implemented guidelines to withdraw medicated feed additives in order to combat antibiotic resistance stemming from animal sources [1]. Metal-containing compounds have been widely used in various applications, including as food additives and nutritional sources, in food production and processing [2]. Furthermore, metals, such as copper, zinc, and cobalt, are widely used in animal feed for their growth-stimulating and antimicrobial properties [2,3,4]. However, the use of heavy metals at high concentrations poses significant risks due to their toxicity, bioaccumulation, and biomagnification within the food chain [2,5,6]. The abundance of heavy metals in common foods, such as fresh vegetables, fruits, and meat, along with the widespread presence of these pollutants in the environment, poses a risk to both food safety and human health [3,7,8,9]. To protect human and animal health, the FDA’s Center for Veterinary Medicine (CVM) operates a Mineral Surveillance Program to actively monitor levels of minerals and heavy metals in animal feeds and food products, ensuring safety and regulatory compliance [10].

Additionally, the presence of heavy metals in food and the environment has led many bacteria to evolve diverse mechanisms of metal resistance through natural selection [11,12]. Potentially more serious, the selective pressure exerted by heavy metals on bacteria can contribute to the selection and spread of AMR to humans through the animal food chain since heavy metal resistance (HMR) and AMR are often linked, leading to potential co-selection. These linkages can occur either through the co-location of resistance genes in mobile genetic elements (MGEs) or through co-regulation of common resistance pathways [2,12,13]. The role of heavy metals and antibiotics in the development and spread of AMR is increasingly acknowledged as a crucial driving force for the selection and spread of AMR in the human and animal food chain [12,14].

As one of the most common causes of foodborne disease in animals and humans worldwide, *Salmonella enterica* is responsible for over 93 million cases of gastroenteritis and 155,000 deaths annually, thus illustrating the global burden of Salmonellosis [15]. In the United States (U.S.), the Centers for Disease Control and Prevention (CDC) estimates that *Salmonella* cause about 1.35 million infections including 26,500 leading to hospitalization, and 420 deaths each year. Most diseases caused by non-typhoidal *Salmonella* result in mild-to-moderate gastroenteritis, typically presenting with abdominal cramps, diarrhea, vomiting, and fever, which begin 12 to 72 h after ingestion of the contaminated food. Patients usually recover in four to seven days, and most improve without the need for antibiotic treatment [16]. In severe cases that can result in more invasive disease, hospitalization and antibiotic therapy are often necessary. These more severe cases most often occur in the very young, elderly, or immunocompromised individuals. Unfortunately, in many cases, antibiotic treatment has become compromised due to the development of AMR [17]. AMR in *S*. *enterica* has become a public health concern due to the rapid spread of the multidrug-resistant (MDR) strains, which cause approximately 410,000 infection every year in the U.S. [18]. The majority of the acquired antimicrobial resistance genes in enteric bacteria are associated with plasmids [19,20]. Key examples include aminoglycoside-resistance genes from the *aac*, *ant*, and *aph* gene families, extended-spectrum beta-lactamases (ESBLs)-resistance genes such as *bla*_CTX-M_, *bla*_SHV_, and *bla*_TEM_ variants, AmpC beta-lactamases-resistance gene families including *bla*_CMY_ and *bla*_DHA_, and carbapenems resistance genes such as *bla*_KPC_, *bla*_NDM_, *bla*_OXA-48_ and *bla*_IMP_ variants, all of which are predominantly plasmid-mediated in *Salmonella* [19,21].

The presence of ARGs on plasmids facilitates their spread among different bacterial species. Similarly to ARGs, some heavy metal resistance genes (HMRGs) are also located on MGEs such as plasmids, transposons, or integrative conjugative elements and they are genetically linked to ARGs [22]. *Salmonella* can harbor HMRGs that are linked to antibiotic and disinfectant resistance genes. Studies have shown that extensive use of heavy metals can lead to the co-selection of multidrug and heavy-metal-resistant *Salmonella,* resulting in co-resistance, where bacteria develop resistance to both heavy metals and antimicrobials simultaneously [22].

Even with this concern for the co-selection of resistance, the prevalence of these HMRGs in *Salmonella* and their impact on the induction of AMR remain unclear. Further, there is limited knowledge on the genetic association of specific HMRGs with specific ARGs. To begin to address these data gaps, this project was undertaken to assess the prevalence of HMRGs in available data from the United States and assess their associations with ARGs in *S. enterica* strains by analyzing whole genome sequence (WGS) data available in NCBI (https://www.ncbi.nlm.nih.gov). The WGS data were collected for strains that originated from various *Salmonella* serotypes, sources (including food, animals, animal feed, and the environment), across different locations and time periods. The findings of this study demonstrated that HMRGs were common among multiple serotypes and strains that carried HMRGs typically displayed higher numbers of ARGs than those strains lacking HMRGs. Many of the HMRGs, especially those associated with mercury resistance, and ARGs were shown to be co-located on diverse types of plasmids, highlighting the widespread nature of the resistance challenges. Overall, this project provided valuable data into the potential concerns of heavy metal usage and resistance and their potential role in cross-resistance to antimicrobials.

## 2. Materials and Methods

### 2.1. Data Sources

**Figure 1:**
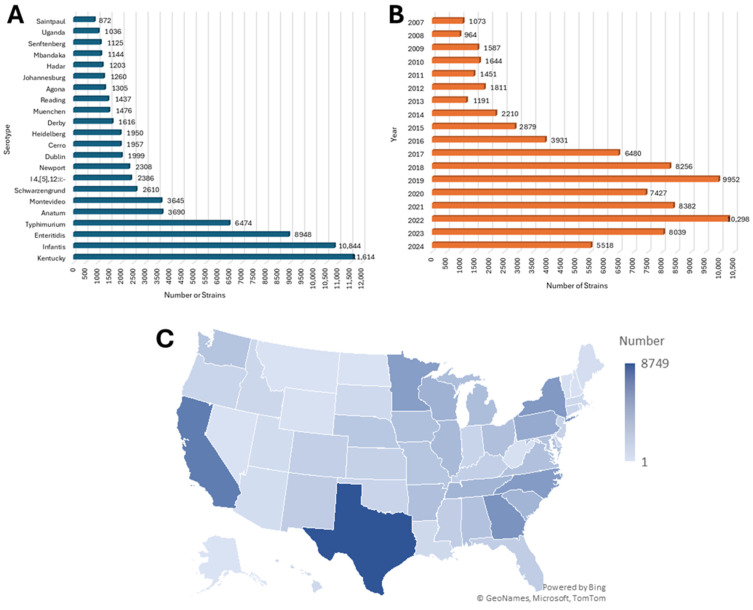
Overview of the demographic distribution of the 86,968 *S. enterica* strains included in study. Panel (**A**) shows a distribution of the most detected serotypes that were observed in the study. There were 16,069 isolates from less prevalence serotypes that are not part of top serovars displayed. Panel (**B**) shows the distribution of strains based on year of isolation. The majority of strains were isolated in 2007 and beyond; however, 3875 were isolated prior to 2007 and their distribution is not included in the figure. Panel (**C**) shows a map density plot of the strains isolated from the different States, with the highest number coming from Texas (N = 8749) ranging down to 23 from Alaska (there were 12 from the District of Columbia). The density scale is shown to the right of the panel.

To investigate the overall prevalence of HMRGs in *Salmonella* isolates, the metadata for all available *Salmonella* isolates with WGS data that were available from the Isolate Browser of NIH National Center for Biotechnology Information (NCBI) Pathogen Detection database were downloaded (https://www.ncbi.nlm.nih.gov/pathogens; accessed on 20 October 2024) [23]. The dataset was provided as a delimited text file and encompassed a diverse range of important attributes such as HMRGs (“Stress genotypes”), ARGs (“AMR genotypes”), strain identification (“Strain”), serovar classification (“Serovar”), isolation sources (“Isolation Sources”), geographical location (“Location”), and collection date (“Collection Date”). The resultant search led to a total of 694,0554 sequences. As the global distribution of data were widely variable (i.e., some countries poorly represented and others with large numbers), this manuscript was chosen to focus on the data of isolates from the United States, where the strains were widely distributed across time and place (Figure 1). There was a total of 86,968 *Salmonella* isolates investigated in this study, which included all strains from the U.S. that had the needed metadata noted above in the selection criteria. Stress genotypes and AMR genotypes were determined using AMRFinderPlus (ver. 3.11.26) results that were pre-calculated by the NCBI during their data curation processes and the resistance genotype data were downloaded from the Pathogen Detection’s Isolates Browser [23]. These data along with the corresponding GenBank accession numbers are provided in the Supplemental Tables. The resistance gene data points provided a holistic foundation for analyzing the patterns in HMRGs and ARGs across wide range of contexts and factors. To investigate prevalence of co-localization of ARGs and HMRGs on plasmids, the PLSDB plasmid database [24] was searched and 72,556 plasmids with metadata indicating they originated from *Salmonella* and contained information on ARGs, HMRGs, and plasmid replicons were identified (accessed on 10 May 2025). Data including their replicon types and the predetermined ARG and HMRG profiles were downloaded from PLSDB.

### 2.2. Data Processing

Once the raw dataset was acquired, extensive processing was necessary to ensure data integrity and analytical accuracy. Microsoft Excel and Python (ver. 3.12). Data Analysis Library (PANDAS) [25] were used to clean, filter, and structure the data. First, the dataset from NCBI Pathogen Detection database was filtered in Microsoft Excel to include only isolates with complete information on serovar, isolation sources, location (state), and collection year. Any entries from countries outside of the U.S. were also excluded to maintain consistency and focus on domestic trends. The dataset was then standardized to further ensure conformity in naming conventions and classifications, further eliminating inconsistencies that could confound results. Then, the filtered metadata were subsequently normalized and analyzed using Excel and PANDAS (ver. 2.2.3). The presence of HMRGs and ARGs were converted to a binary such that a ‘1′ indicates reported presence and a ‘0′ indicates absence of that gene. In the original metadata file, these genes were listed in a single column, with the presence of each gene marked as ‘= complete‘ or ‘= HMM‘. Similarly, the metadata of the plasmids downloaded from the PLSDB plasmid database were filtered using PANDAS to include only plasmids that contained at least one of the ARGs or HMRGs associated with the AMRFinderPlus dataset in the Isolates Browser. The data were further processed into a binary format so that a value of ‘1′ indicated the presence of a gene, and ‘0′ indicated its absence.

### 2.3. Analysis of HMRG Prevalence in Salmonella Isolates

The HMRGs studied and their products are listed in Table 1. To further investigate potential correlations between HMRGs and ARGs, *Salmonella* isolates were divided into two groups: those containing HMRGs and those without. The frequency and number of ARGs were then compared between these two groups. Specifically, the percentage of isolates with 0, 1, 2, 3–5, 6–10, 11–20, and 21–29 ARGs was calculated for each group. Also, the percentage of ARGs in isolates with and without key HMRGs (*pcoA*, *silA*, *merA*, and *arsB*) were calculated and compared. For each ARG, the percentage of occurrence in HMRG-negative isolates was subtracted from that in HMRG-positive isolates. A more negative difference indicated that the ARG was more commonly found in the absence of HMRGs, while a more positive difference suggested a greater likelihood of co-occurrence between the ARG and HMRGs.

### 2.4. Screening of Plasmids for ARGs and HMRGs

To investigate prevalence of co-localization of ARGs and HMRGs on plasmids, the data downloaded data from the PLSDB were analyzed to determine the presence of ARGs, HMRGs, and plasmid replicons. The percentages of the plasmids that contain at least one ARG and one HMRG were calculated using Microsoft Excel. The plasmids with and without co-localization of the ARGs and HMRGs were compared to see their difference in plasmid incompatibility types.

### 2.5. Statistical Analyses

The majority of the statistical analyses conducted involved descriptive statistics, including the prevalence and percentages of individual resistance genes in isolates from different serotypes, sources, locations (states), and isolation times, were calculated using a combination of Microsoft Excel and PANDAS. To assess whether the number of ARGs differ significantly between HMRG-positive and HMRG-negative isolates, chi-square tests were conducted separately for positive and negative isolates for each of the following HMRGs: *pcoA*, *silA*, *merA*, and *arsB*. For each gene, isolates were grouped into ARG count bins (0, 1, 2, 3–5, 6–10, 11–20, and 21–29), and the counts in each group were compiled into contingency tables. To further assess whether the presence of ARGs is significantly associated with HMRG status, Chi-square tests of independence were performed for each of the four HMRGs. For each test, contingency tables were constructed based on the number of isolates in which each ARG was present or absent among HMRG-positive and HMRG-negative groups. Chi-square tests were then performed using the chi2_contingency function from SciPy stats library (https://docs.scipy.org/doc/scipy/reference/generated/scipy.stats.chi2_contingency.html; accessed on 10 September 2025) [26].

## 3. Results and Discussion

### 3.1. Demographic Characteristics of Salmonella Isolates

In total, 86,968 *Salmonella* isolates were investigated in this study. Among them, a majority (80.58%) of the isolates were sourced from animals (N = 70,081), followed by 8.80% from food (N = 7649), 6.01% from human patients (N = 5230), and 4.50% from environmental sources (N = 3916). Fewer than 0.1% of isolates were from other sources, including animal feed (N = 69), eggs (N = 12) or laboratory strains (N = 11). In terms of serotypes, the top five serotypes with the highest numbers of isolates were Kentucky (N =11,614, 13.35%), Infantis (N = 10, 844, 12.47%), Enteritidis (N = 8948, 10.29%), Typhimurium (N = 6474, 7.44%), and Anatum (N =3690, 4.24%) (Figure 1A). Of the strains in study, 95.54% were isolated from 2007 to 2024 (Figure 1B), while the remaining strains (N = 3875) originated prior to 2007 (predominantly between 1981 and 2006). In terms of geographic origin, the five states with the highest numbers of isolates were Texas (N =8749, 10.06%), California (N = 6289, 7.23%), Georgia (N = 4900, 5.63%), New York (N = 4549, 5.23%), and North Carolina (N = 4385, 5.04%) (Figure 1C).

### 3.2. Prevalence of HMRGs Across Different Serotypes of Salmonella

**Figure 2:**
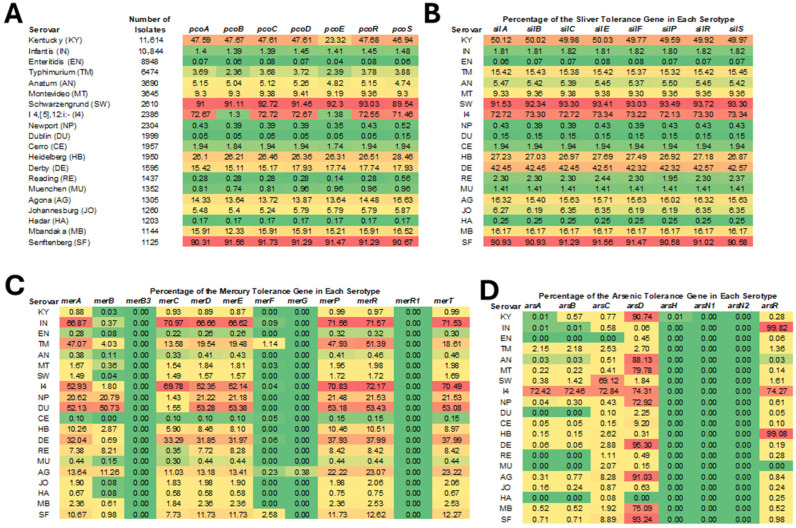
Heatmap of HMRG prevalence across the top *Salmonella* serotypes. Each cell represents the percentage of isolates in each serotype carrying the indicated gene. Panel (**A**) provides the key for the serotypes and number of strains in each group, along with the data on the copper resistance genes. The specific predicted functions for the gene products are shown in Table 1. Panels (**B**–**D**) provide the percent positive data for silver, mercury and arsenic HMRGs, respectively. The color scheme in each panel is spectrum ranging from green (none detected) through yellow and orange (mid-range) to red (highest percent detected).

The prevalence of the HMRGs varied significantly among different *Salmonella* serotypes. Notably, gold resistance genes *golS*, encoding Au(I) sensor transcriptional regulator, and *golT* encoding gold/copper-translocating P-type ATPase, were detected in over 99% of all isolates and therefore excluded from detailed analysis. Their near ubiquity supports the hypothesis that certain metal resistance traits have become genomically entrenched in *Salmonella* populations. Figure 2 illustrates the distribution of HMRGs across the top 20 *Salmonella* serotypes, revealing distinct patterns of gene presence. The prevalence rate differences of silver and copper resistance genes among serotypes were generally consistent and high rates were observed in specific serotypes, particularly Kentucky, Schwarzengrund, I 4,[5],12:i:- (except for *pcoB* and *pcoE*), and Senftenberg. Approximately 90% of isolates from serotypes Schwarzengrund and Senftenberg carried these HMRGs, whereas in serotype Enteritidis, their prevalence was below 0.1%.

The *pco* cluster is a plasmid-borne copper resistance system that is involved in copper resistance in bacteria, particularly in *Enterobacteriaceae* like *Escherichia coli* and *Salmonella* [27,28,29]. It contains seven genes, *pcoABCDERS*, that encode protein with different functions in the copper sensing, transport, and sequestration, helping the organism survive in environments with elevated copper levels [28,30]. The functions of the gene products are shown in Table 1. Although not all genes in the *pco* operon are strictly required for copper resistance, the core resistance proteins are encoded by *pcoA*, *pcoB*, *pcoC*, and *pcoD*. The reason for the low prevalence of *pcoB* (only 1.3%) compared to the other 3 core genes in isolates of serotype I 4,[5],12:i:- remains unclear, but this finding is not unique; a previous study reported the presence of *pcoA* in the absence of *pcoB* in 19 out of 268 genomes analyzed [31]. PcoE helps buffer sudden copper increases and has been reported to be important to decreasing the free copper concentration; however, it is not essential for baseline resistance [32]. *pcoR* and *pcoS* encode a two-component regulatory system that detects copper and regulates expression of the *pco* operon. Often co-located with *pco* operon on plasmids is the *sil* operon, which usually consists of eight genes as highlighted in Table 1 [33]. Similar prevalence patterns of the *pco* and *sil* operons among different *Salmonella* serotypes observed in this study support their genetic co-localization [31,33].

The prevalence of different mercury resistance genes varied more widely across serotypes. The mercury resistance operon (*mer* operon) varies in structure and consists of genes that encode proteins with diverse functions as outlined in Table 1 [34,35]. The results from this study show that *merF* and *merG* were rare across all serotypes. Their proteins facilitate the transport of both organic phenylmercury and inorganic Hg^2+^ across the cytoplasmic membrane and contribute to reducing cellular permeability to organomercurial compounds such as phenylmercury by promoting their efflux [32]. As both are considered auxiliary transport proteins, their low prevalence in *Salmonella* is not unexpected. A previous study also suggested that the *merG* gene is more commonly found in the more recently evolved operons [33]. The *merB* had a low detection rate in most serotypes, except for *Salmonella* Dublin (50.73%) and Newport (20.79%), where it showed relatively high prevalence. Isolates carrying both *merA* and *merB* confer broad-spectrum mercury resistance, whereas isolates with *merA* alone confer only narrow-spectrum inorganic mercury resistance [35]. Another study showed that, compared to genes encoding narrow-spectrum mercury resistance (e.g., *merA*), *merB* and *merG* were less frequently detected [36]. The other mercury resistance genes exhibited an elevated prevalence in *S.* Infantis, I 4,[5],12:i:-, Dublin (excluding *merC*), and Derby.

The prevalence of different arsenic resistance genes also varied considerably across serotypes. The arsenic resistance operon (*ars* operon) consists of six genes with varied functions [37,38,39] (Table 1). Our study found that genes *arsA* and *arsB* exhibited similar prevalence patterns across different serotypes, which would be expected at they are part of two-component efflux pump. Both genes were present in approximately 72% of isolates from serotype I 4,[5],12:i:-, but were detected in fewer than 3% of isolates from other serotypes. *arsC* showed relatively high prevalences in only *S.* Schwarzengrund and I 4,[5],12:i:-, with rates of 69.12% and 72.84%, respectively. High detection rates of *arsR* were observed in *S.* I 4,[5],12:i:-, Infantis, and Heidelberg, with rates in *S.* Infantis and Heidelberg exceeding 99%. *arsD* showed the highest overall prevalence among the arsenic resistance genes and was especially common in serotypes Kentucky, Anatum, Montevideo, I 4,[5],12:i:-, Newport, Derby, Agona, Mbandaka, and Senftenberg. In these serotypes, the prevalence rates ranged from 72.92% to 95.30%; conversely, the *arsD* prevalence was below 3% in other serotypes. *arsH* was not detected in any isolates investigated in this study. Previous studies have also shown that the distribution of arsenic resistance genes varied among different serotypes [33,38].

Overall, HMRGs were detected at a higher frequency in isolates of serotype I 4,[5],12:i:- compared to other serotypes (Figure 2). In contrast, *S.* Enteritidis, one of the most clinically significant serotypes, had limited carriage of the *pco*, *sil*, *mer*, or *ars* genes (Figure 2). This disparity may reflect differing ecological pressures or suggest distinct evolutionary trajectories between these isolates. The relatively low prevalence of HMRGs in *S.* Enteritidis may reflect several underlying factors. Genomic characteristics unique to *S.* Enteritidis may constrain the acquisition or stable maintenance of plasmids or genomic islands carrying HMRGs [40]. It is also possible that *S. Enteritidis* employs alternative mechanisms for coping with metal stress that were not detected in this study. Overall, the serotypes that had the highest proportion of copper and silver resistance genes were serotypes that have commonly associated with poultry [40], which may indicate that plasmids that carry the HMRGs are circulating among *Salmonella* in poultry and/or that certain practices are selecting for their maintenance in these environments.

### 3.3. Prevalence of HMRGs Across Different Isolation Sources

**Figure 3:**
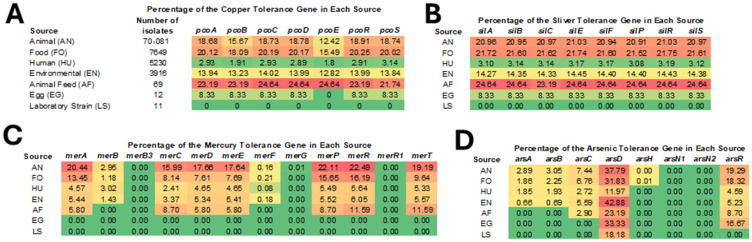
Distribution of HMRGs across isolation sources (animal, human, food, environment, etc.). Panel (**A**) provides the key for the sources and number of strains in each group, along with the data on the percentage of isolates that carry the specific copper resistance genes. The specific predicted functions for the gene products are shown in Table 1. Panels (**B**–**D**) provide the percent positive data for silver, mercury and arsenic HMRGs, respectively. The color scheme in each panel is spectrum ranging from green (none detected) through yellow and orange (mid-range) to red (highest percent detected).

The isolates examined in this study were classified into seven different source groups: animal, food, human, environmental, animal feed, egg, and laboratory. The distribution of sources was heavily weighted to those from animal sources, which is indicative of the volume of isolates sequenced by the U.S. Department of Agriculture’s Food Safety and Inspection Service (USDA-FSIS, as indicated in sequence metadata). Relatively high rates of HMRGs were observed in isolates from animals, food, and animal feed, whereas lower rates were found in human clinical isolates (Figure 3). This pattern may be indicatives of exposures in the animal environments or due to the distribution of serotypes associated with the different sources. For example, *S.* Enteritidis is one of the top serotypes from human patients and, as noted above, has limited numbers of HMRGs. In other instances, strains from animal and food production sectors could likely serve as a reservoir of HMRGs through the food chain to human patients.

### 3.4. Prevalence of ARGs in HMRG-Positive vs. -Negative Isolates

**Figure 4:**
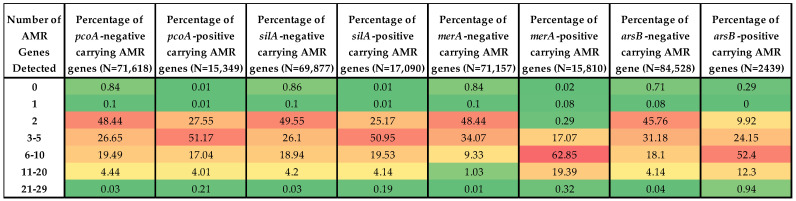
Heatmap of the distribution of AMR gene levels in HMRG-negative vs. HMRG-positive isolates for *pcoA*, *silA*, *merA*, and *arsB*, which serve as representative HMRGs for their corresponding operons. A larger proportion of HMRG-positive isolates carry high numbers (21–29) of AMR genes. The color scheme in each panel is spectrum ranging from green (none detected) through yellow and orange (mid-range) to red (highest percent detected).

To investigate the association between the HMRGs and ARGs, the numbers of ARGs in isolates with and without key HMRGs (*pcoA*, *silA*, *merA*, *arsB*) were counted and compared. The results showed that, for all four HMRGs, positive isolates carried significantly more ARGs compared to the corresponding HMRG-negative isolates (Figure 4). For example, among *pcoA*-positive isolates, 51.17% carried 3–5 ARGs, compared to only 26.65% of *pcoA*-negative isolates. A similar pattern of elevated numbers of ARGs among the *silA*, *merA*, and *arsB* positive isolates was observed in the dataset as shown in Figure 4.

Chi-square test results revealed statistically significant differences in the distribution of ARGs between HMRG-positive and HMRG-negative isolates for all four genes examined (*p* < 0.0001 for each, excepted where specifically noted; Table 2). This indicates that the presence of *pcoA*, *silA*, *merA*, or *arsB* is associated with varying levels of ARG burden among bacterial isolates. These findings highlight a strong correlation between HMRG presence and elevated antimicrobial resistance, suggesting possible co-selection or co-localization of HMRGs and ARGs on MGEs. Several ARGs were found to be strongly associated with certain HMRGs. The prevalence of *tet(A)*, *sul1*, *sul2*, *aadA1*, *floR*, *aac(3)-IVa*, and *aph(4)-Ia* was notably higher in *merA*-positive isolates compared to *merA*-negative ones. while *aph(3″)-Ib*, *aph(6)-Id*, and *tet(B)* showed higher prevalence in isolates positive for *pcoA*, *silA*, and *arsB* than in their corresponding negative groups. In addition to *merA*, *sul2* also shows a strong association with *arsB*, a relationship that has also been reported in a previous study [37]. The findings from this study suggested that these ARGs may be co-selected or co-localized with specific HMRGs, potentially residing on shared MGEs such as plasmids or transposons, facilitating their joint dissemination under selective pressures like heavy metal exposure or antibiotic use.

### 3.5. Co-Localization of ARGs and HMRGs

**Figure 5:**
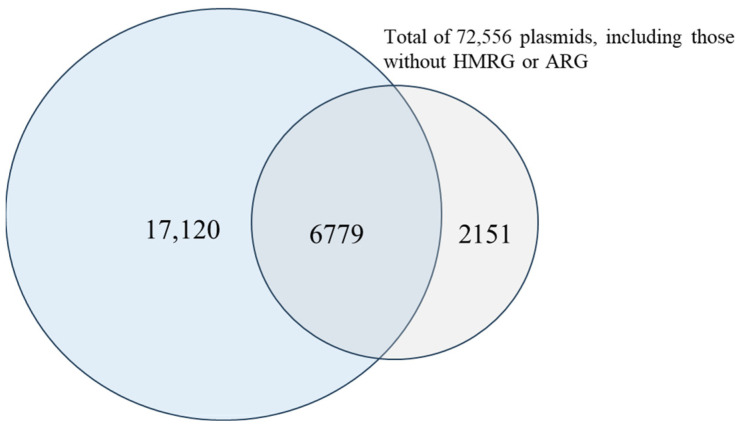
Venn diagram showing the plasmids with ARGs (light blue), the plasmids with HMRGs (light gray), and their intersection (the plasmids with both ARGs and HMRGs).

Previous results from this study revealed a strong association between HMRGs and ARGs through analysis of WGS data. Since many of these genes are plasmid-encoded, further analysis of their presence on plasmids could provide valuable insights into their potential for horizontal gene transfer and the co-selection mechanisms that drive the spread of both heavy metal and antimicrobial resistance. A total of 72,556 plasmids with associated metadata were available in the PLSDB plasmid database as of 10 May 2025. These included plasmids from *S. enterica* and other species, since many plasmids have host ranges that span across species barriers. Among them, 32.94% (N = 23,899) carried at least one ARGs, 12.31% (N = 8930) carry at least one HMRG, and 9.34% (N = 6779) contained both ARG and HMRG (Figure 5). Metadata for 26,050 plasmids that contained at least one of the HMRGs or ARGs were downloaded for this study.

**Figure 6:**
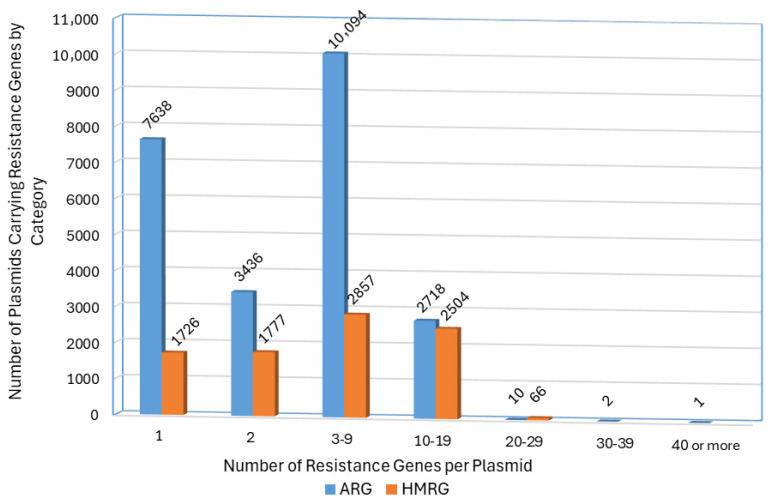
Distribution of numbers of ARGs and HMRGs counts per plasmid in those plasmids that carried at least one resistance gene. There was a total of 23,899 plasmids that carried at least one ARG (blue bars) and 6779 plasmids that carried at least one HMRG (orange bars).

Among the 23,899 plasmids carrying at least one ARG, 31.96% (N = 7638) contained a single ARG, 14.38% (N = 3436) contained two ARGs, while the remainder harbored multiple genes, sometimes encoding resistance to several different classes of antibiotics (Table S1, Supplemental Data). The distribution of plasmids based on the number of ARGs they carry was presented in Figure 6. When we look specifically among the 1568 plasmids that were isolated from *Salmonella* sources, 22.32% (N = 350) had one ARG, 7.84% (N = 123) carried two ARGs, and the remaining 69.04% carried more than two ARGs. These observations would indicate that the *Salmonella*-associated plasmid were skewed to having more ARGs than the broader plasmid population. The most commonly observed resistance gene classes that were present in more than 20% of all of the plasmids included *bla* genes (β-lactams; N = 15,203, 63.61%), *sul* (sulfonamide; N = 9175, 38.39%), *aad* (aminoglycoside; N = 7144, 29.89%), *tet* (tetracycline; N = 7027, 29.40%), *qac* (quaternary ammonium compounds; N = 7018, 29.37%), *aph* (aminoglycoside; N = 6786, 28.39%), *aac* (aminoglycoside; N = 6191, 25.90%), and *dfrA* (trimethoprim; N = 6159, 25.77%). These genes were also the predominant ARGs among the *Salmonella*-originating plasmids (Table S1, Supplemental Data).

There were 69 unique HMRGs for arsenic, cadmium, chromate, copper, gold, mercury, nickel, potassium, silver, or tellurium identified among the 8930 HMRG-carrying plasmids (Table S2, Supplemental Data). The most common HMRGs for mercury (N = 4137; 46.33%), followed by copper (N = 2955; 33.09%;), silver (N = 2826; 31.65%), tellurium (N = 2338; 26.18%) to arsenic (N = 1938; 21.70%), and cadmium (N = 1080; 12.09%). Resistance genes for the remaining four heavy metals (chromate, gold, nickel, and potassium) were present in less than 0.50% of the plasmids. Among the HMRG-carrying plasmids, 19.16% (N = 1711) contained a single identified HMRG, 20.02% (N = 1788) contained two HMRGs, while the remaining plasmids (N = 5431; 60.82%) harbored multiple genes that may confer resistance to different heavy metals. The distribution of HMRG counts among plasmids is presented in Figure 6. Among the 764 *Salmonella*-isolated plasmids with HMRGs, there were some differences among the most common HMRGs, with the mercury resistance gene, *merT,* present in 58.24% (N = 445) of the plasmids, followed by tellurium resistance genes in up to 45.68% (N = 349), and then copper resistance genes in approximately 13% of the plasmids. The copper, arsenic, and silver HMRGs were present in much lower prevalence (>10% lower) among the *Salmonella*-associated plasmids (Table S2, Supplemental Data). Tellurium and some mercury resistance genes are common in IncHI2 plasmids [41,42], which were commonly detected plasmid replicon types among the *Salmonella*-associated plasmids in this study (Table S3, Supplemental Data).

To assess the commonalities among AMR and HMR factors, the average percentage of plasmids that carried both types of resistance determinants was identified. The results showed that commonly used antibiotics (aminoglycoside, beta-lactam, chloramphenicol, macrolide, sulfonamide, tetracycline, and trimethoprim) all exhibited certain commonalities with HMRG classes (Table 3). For example, the mercury resistance operon exhibited significant overlap with genes conferring resistance to aminoglycosides, beta-lactam, and sulfonamide which is consistent with the results from previous studies [38]. These results highlight that mercury resistance genes are often co-located with ARGs in transposons common in many plasmids [43].

Among the HMRG and ARG carrying plasmids, 80.04% (N = 5426) were assigned to at least one known plasmid incompatibility (Inc) type (Table S3, Supplemental Data). Of these plasmids, 55.68% (N = 3021) were shown to display more than one incompatibility type. The five most frequently identified Inc types were IncFIB (N = 2315; 42.66%), IncFII (N = 1617; 30.80%), IncHI2A (N = 1072; 19.76%), IncFIA (N = 874; 16.11%), and IncR (N = 690; 12.72%). Each of these plasmid types were commonly detected within 723 *Salmonella*-isolated plasmids with a reported Inc type (Table S3, Supplemental Data), with the exception of IncR. IncR plasmids, along with the other plasmid types have been reported in the literature to be commonly associated with the Enterobacteriaceae, including *Salmonella* [42,44]. The IncC plasmids were common among the *Salmonella*-associated plasmids (17.29%, N = 125) compared to the larger group of plasmids (9.01%, N = 489). IncC (often previously characterized as IncA/C plasmids) are prominent AMR plasmids that typically harbor transposons carrying mercury resistance genes [41,45]. Further analysis was conducted to determine whether there were differences in plasmid incompatibility types between plasmids with and without co-localization of ARGs and HMRGs. As shown in Table 4, the distribution of plasmid incompatibility types among plasmids carrying both mercury resistance and ARGs (beta-lactam or aminoglycoside resistance) were similar to one another and to plasmids lacking these ARGs. This suggests no specific incompatibility group appeared to be uniquely associated with the co-localization of HMRGs and ARGs, indicating that the co-selection of resistance traits may occur across a broad range of plasmid types rather than being confined to a particular incompatibility group.

## 4. Conclusions

HMRGs are widely distributed among *Salmonella* isolates, with notable variation in prevalence among different serotypes and isolation sources. The observed correlation between the presence of HMRGs and higher ARG counts supports the hypothesis that co-selection pressures, such as environmental exposure to heavy metals, may contribute to the persistence and spread of multidrug-resistant *Salmonella* strains. Several ARGs, including *tet(A)*, *sul1*, *aadA1*, and *floR*, were significantly more prevalent in HMRG-positive isolates, supporting potential co-localization on shared MGEs such as transposons and their associated plasmids [41]. The diversity of plasmids carrying both HMRGs and ARGs was notable, suggesting the likely sharing of genetic material among different types of plasmids that may be co-located in bacteria, a fairly common phenomenon observed in *Salmonella* [43,45]. This sharing and plasmid genetic mixing were highlighted in the current study, as over half of the assembled plasmids analyzed had more than one replicon (incompatibility) type identified for them, underscoring the complexity and modular nature of plasmid-mediated resistance. However, no specific incompatibility type was uniquely associated with the co-localization of ARGs and HMRGs, suggesting that such co-resistance can disseminate broadly across diverse plasmid backbones via MGEs such as transposons and integrons. The importance of integrated surveillance and the further incorporation of demographic data and comparative genomics across clinical, agricultural, and environmental settings will lead to better approaches to mitigate the risk of multidrug resistance dissemination via MGEs in the food supply.

## Figures and Tables

**Table 1:** The list of HMRGs and their predicted products that were investigated in this study.

Metal Resistance	Metal Resistance Genes	Products
Mercury	*merA*	mercury(II) reductase
	*merB*	organomercurial lyase MerB
	*merC*	organomercurial transporter MerC
	*merD*	mercury resistance co-regulator MerD
	*merE*	broad-spectrum mercury transporter MerE
	*merP*	mercury resistance system periplasmic binding protein MerP
	*merR*	mercury resistance transcriptional regulator MerR
	*merT*	mercuric transport protein MerT
Arsenic	*arsA*	arsenite efflux transporter ATPase subunit ArsA
	*arsB*	arsenite efflux transporter membrane subunit ArsB
	*arsC*	glutaredoxin-dependent arsenate reductase
	*arsD*	arsenite efflux transporter metallochaperone ArsD
	*arsH*	organoarsenical oxidase ArsH
	*arsR*	arsenite efflux transporter ATPase subunit ArsA
Sliver	*silA*	Cu(+)/Ag(+) efflux RND transporter permease subunit SilA
	*silB*	Cu(+)/Ag(+) efflux RND transporter periplasmic adaptor subunit SilB
	*silC*	Cu(+)/Ag(+) efflux RND transporter outer membrane channel SilC
	*silE*	silver-binding protein SilE
	*silF*	Cu(+)/Ag(+) efflux RND transporter periplasmic metallochaperone SilF
	*silP*	Ag(+)-translocating P-type ATPase SilP
	*silR*	copper/silver response regulator transcription factor SilR
	*silS*	copper/silver sensor histidine kinase SilS
Copper	*pcoA*	multicopper oxidase PcoA
	*pcoB*	copper-binding protein PcoB
	*pcoC*	copper resistance system metallochaperone PcoC
	*pcoD*	copper resistance inner membrane protein PcoD
	*pcoE*	copper resistance system metallochaperone PcoE
	*pcoR*	copper response regulator transcription factor PcoR
	*pcoS*	copper resistance membrane spanning protein PcoS
Gold	*golS*	gold-responsive transcriptional regulator GolS
	*golT*	gold-exporting ATPase GolT

**Table 2:** The percentage difference in prevalence of each AMR gene between isolates with and without the listed key metal resistance gene, all differences had *p*-values < 0.0001 unless noted in parentheses next to corresponding χ^2^ Value.

AMR Gene	*pcoA*	χ^2^ Value	*silA*	χ^2^ Value	*merA*	χ^2^ Value	*arsB*	χ^2^ Value
*tet(A)*	−17.93	2322.81	−12.85	1293.79	78.51	45,529.57	−17.84	429.93
*aph(3′’)-Ib*	38.26	10,915.40	35.31	10,092.07	3.39	87.63	44.32	2744.77
*aph(6)-Id*	38.3	11,063.08	35.43	10,283.84	3.33	85.56	44.45	2793.32
*sul1*	−9.36	881.17	−5.29	304.62	58.48	35,273.56	−5.05	47.64
*tet(B)*	25.4	7122.68	26.21	8231.89	−2.65	79.20	67.64	9467.24
*sul2*	1.97	46.55	5.41	382.57	37.37	17,223.18	51.79	6058.76
*gyrA_D87Y*	−14.32	2477.14	−14.68	2833.31	42.45	22,300.25	−12.16	334.12
*aadA1*	−8.01	776.69	−4.88	312.39	52.17	33,742.17	−7.29	119.83
*floR*	−9.99	1409.29	−9.63	1425.33	30.94	13,835.70	−5.16	69.95
*fosA7*	−1.25	33.05	−1.76	71.34	−5.9	757.21	−6.01	143.55
*aac(3)-IVa*	−4.69	473.42	−4.88	553.33	25.51	14,315.98	−3.37	45.19
*aph(4)-Ia*	−4.73	483.26	−4.92	569.14	25.51	14,367.89	−3.56	50.74
*bla*CMY-2	−3.26	247.38	−3.38	291.70	15.04	5433.14	−3.12	42.47
*bla*TEM-1	9.06	1971.50	9.1	2160.76	8.18	1644.41	59.06	15,722.66
*bla*CTX-M-65	−4.14	622.11	−4.27	719.19	16.25	9782.97	−3.56	85.16
*dfrA14*	−3.46	448.97	−3.58	522.57	13.94	7493.32	−1.97	26.90
*aadA2*	0.06	0.09 (*p* = 0.77)	1.15	65.84	5.64	1506.24	5.21	234.30
*aac(3)*-VIa	0.74	35.07	4.37	1348.27	5.75	2183.20	−0.15	0.22
*fosA7.3*	−0.68	33.34	2.3	418.68	1.83	251.72	−1.42	27.02
*tet(C)*	−0.37	10.84 (*p* = 0.001)	3.19	840.32	2.73	568.85	−0.4	2.01
*fosA7.2*	−0.4	13.12 (*p* = 0.0003)	−0.37	12.53 (*p* = 0.0004)	−0.42	14.06	−1.18	21.34
*fosA3*	−1.77	274.54	−1.81	312.69	6.76	4104.97	−1.5	36.09
*qnrB19*	2.79	724.29	2.83	808.80	0.85	68.09	11.58	2334.25
*bla*TEM	−1.07	114.21	−1.11	132.26	5.03	2579.47	−0.09	0.09 (*p* = 0.76)
*bla*CARB-2	−1.21	172.27	−0.93	109.44	−0.52	33.63	−1.05	23.86
*tet(G)*	−1.11	170.73	−0.93	129.84	−0.42	24.77	−0.94	22.12

Note: A positive number indicates that the prevalence of the AMR gene is higher in isolates with the metal resistance gene, whereas a negative number suggests the opposite.

**Table 3:** Percentage of plasmids with HMRG classes that carry ARGs for different antimicrobial resistance classes.

HMRG Classes on Plasmid	AG	BL	BLE	CHL	EP	FOF	LNC	ML	MUP	OXL	PMB	QAC	QL	RIF	STP	SUL	TET	TMP
Arsenic	14.46	12.85	0.68	9.51	0.43	0.27	0.03	5.68	0.01	0.03	0.24	7.35	6.25	1.00	0.04	12.70	7.23	10.95
Copper	22.98	18.65	1.25	14.80	0.99	0.28	0.81	9.66	0.00	0.25	0.50	12.60	9.78	3.08	0.83	18.94	12.13	16.48
Mercury	44.41	44.92	4.84	24.28	1.96	3.27	0.89	17.42	0.00	0.03	2.45	31.24	12.18	7.69	0.18	37.82	23.48	24.03
Silver	21.68	18.51	1.77	14.25	1.34	0.25	0.35	8.73	0.00	0.00	0.34	12.23	9.74	1.77	0.01	18.82	12.55	15.49
Cadmium	4.99	13.54	0.03	0.03	0.19	0.09	0.06	5.24	0.19	0.00	0.00	1.40	0.00	0.00	3.91	0.00	0.55	0.43
Tellurium	22.33	20.17	5.83	14.74	4.41	2.77	1.83	10.75	0.00	0.00	2.86	16.46	10.71	7.64	0.12	20.27	12.49	14.04

Antimicrobial Class Abbreviations: Aminoglycoside (AG), Beta-lactam (BL), Bleomycin (BLE), Chloramphenicol (CHL), Efflux pump (EP), Fosfomycin (FOF), Lincosamide (LNC), Macrolide (ML), Mupirocin (MUP), Oxazolidinone (OXL), Polymyxin B (PMB), Quaternary ammonium compound (QAC), Quinolone (QL), Rifampin (RIF), Streptothricin (STP), Sulfonamide (SUL), Tetracycline (TET), and Trimethoprim (TMP). The color scheme in each panel was used to highlight prevalence differences, the spectrum ranging from green (none detected) through yellow and orange (mid-range) to red (highest percent detected). Note: ARG classes found on less than ten plasmids were excluded from the table.

**Table 4:** Distribution of plasmid replicon types among typable plasmids either co-localizing mercury and beta-lactam (BL, blue), mercury and aminoglycoside (AM, peach), or those plasmids carrying mercury HMRGs, but not BL or AM ARGs (yellow).

Plasmid Replicon Type	Co-Localized Mercury and Beta-Lactam ARGs (N = 2729)		Co-Localized Mercury and Aminoglycoside ARGs (N = 2584)		Mercury Resistance Genes, but not Aminoglycoside or Beta-Lactam ARGs (N = 785)	
	Number	Percent	Number	Percent	Number	Percent
IncFIB	870	31.88	843	32.62	223	28.41
IncFII	690	25.28	666	25.77	168	21.4
IncFIA	638	23.38	597	23.1	184	23.44
IncR	504	18.47	461	17.84	159	20.25
IncHI2A	420	15.39	383	14.82	119	15.16
IncU	382	14	355	13.74	123	15.67
IncC	364	13.34	358	13.85	111	14.14
IncQ1	207	7.59	220	8.51	57	7.26
IncFIC	155	5.68	149	5.77	39	4.97
IncHI1B	153	5.61	145	5.61	58	7.39
IncN	86	3.15	72	2.79	29	3.69
IncP	82	3	77	2.98	23	2.93
IncL/M	55	2.02	54	2.09	16	2.04
IncK2/Z	48	1.76	46	1.78	9	1.15
IncI-gamma/K1	41	1.5	34	1.32	14	1.78
IncHI1A	37	1.36	41	1.59	9	1.15
IncX1	34	1.25	33	1.28	10	1.27
IncY	26	0.95	26	1.01	9	1.15
IncA	23	0.84	21	0.81	11	1.4
IncX3	11	0.4	10	0.39	1	0.13
Col(VCM04)	10	0.37	10	0.39	3	0.38
IncT	6	0.22	8	0.31	1	0.13
Col3M	3	0.11	3	0.12	1	0.13
Col156	2	0.07	2	0.08	1	0.13
Inc18	2	0.07	2	0.08	0	0
IncQ2	2	0.07	1	0.04	1	0.13
ColE10	1	0.04	0	0	1	0.13
IncI1	1	0.04	1	0.04	0	0
IncI1/B/O	1	0.04	1	0.04	0	0
IncW	1	0.04	1	0.04	0	0
IncX4	1	0.04	0	0	1	0.13
IncX3	0	0	0	0	1	0.13

## Data Availability

The original contributions presented in this study are included in the article/Supplementary Materials. Further inquiries can be directed to the corresponding author.

## Supplementary Materials

The following supporting information can be downloaded at: https://www.mdpi.com/article/10.3390/microorganisms13122696/s1, Table S1: Table containing the ARG profiles for all the plasmids analyzed from different bacterial sources that contained at least one ARG. The data are presented in binary format, with “1” meaning the gene was detected and “0” indicating that it was not detected. Table S2: Table containing the HMRGs profiles for all the plasmids containing at least one HMRG in the study. The data are presented in binary format, with “1” meaning the gene was detected and “0” indicating that it was not detected. Table S3: Table containing the different plasmid replicon/incompatibility types (across the rows) that were identified for each plasmid that carried at least one HMRG and one ARG. The plasmids with more replicon types detected for each plasmid are positioned at the top of the list. In each of the supplemental tables, the plasmids that originated from *Salmonella* strains are displayed at the tops of the respective tables.

## References

[1] Hu Y.J., Cowling B.J. (2020). Reducing antibiotic use in livestock, China. Bull. World Health Organ..

[2] Mustafa G.R., Zhao K., He X., Chen S., Liu S., Mustafa A., He L., Yang Y., Yu X., Penttinen P. (2021). Heavy Metal Resistance in Salmonella Typhimurium and Its Association with Disinfectant and Antibiotic Resistance. Front. Microbiol..

[3] Argudin M.A., Lauzat B., Kraushaar B., Alba P., Agerso Y., Cavaco L., Butaye P., Porrero M.C., Battisti A., Tenhagen B.A. (2016). Heavy metal and disinfectant resistance genes among livestock-associated methicillin-resistant *Staphylococcus aureus* isolates. Vet. Microbiol..

[4] Lemire J.A., Harrison J.J., Turner R.J. (2013). Antimicrobial activity of metals: Mechanisms, molecular targets and applications. Nat. Rev. Microbiol..

[5] Balali-Mood M., Naseri K., Tahergorabi Z., Khazdair M.R., Sadeghi M. (2021). Toxic Mechanisms of Five Heavy Metals: Mercury, Lead, Chromium, Cadmium, and Arsenic. Front. Pharmacol..

[6] Luo L., Wang B., Jiang J., Fitzgerald M., Huang Q., Yu Z., Li H., Zhang J., Wei J., Yang C. (2020). Heavy Metal Contaminations in Herbal Medicines: Determination, Comprehensive Risk Assessments, and Solutions. Front. Pharmacol..

[7] Sarker A., Kim J.E., Islam A., Bilal M., Rakib M.R.J., Nandi R., Rahman M.M., Islam T. (2022). Heavy metals contamination and associated health risks in food webs-a review focuses on food safety and environmental sustainability in Bangladesh. Environ. Sci. Pollut. Res. Int..

[8] Lu Y., Song S., Wang R., Liu Z., Meng J., Sweetman A.J., Jenkins A., Ferrier R.C., Li H., Luo W. (2015). Impacts of soil and water pollution on food safety and health risks in China. Environ. Int..

[9] Lin L., Yang H., Xu X. (2022). Effects of Water Pollution on Human Health and Disease Heterogeneity: A Review. Front. Environ. Sci..

[10] Deemy M., Benjamin L. (2019). CVM CY15-17 Report on Heavy Metals in Animal Food.

[11] Vats P., Kaur U.J., Rishi P. (2022). Heavy metal-induced selection and proliferation of antibiotic resistance: A review. J. Appl. Microbiol..

[12] Yang S., Deng W., Liu S., Yu X., Mustafa G.R., Chen S., He L., Ao X., Yang Y., Zhou K. (2020). Presence of heavy metal resistance genes in *Escherichia coli* and *Salmonella* isolates and analysis of resistance gene structure in *E. coli* E308. J. Glob. Antimicrob. Resist..

[13] Capita R., Alonso-Calleja C. (2013). Antibiotic-resistant bacteria: A challenge for the food industry. Crit. Rev. Food Sci. Nutr..

[14] Tan Y., Zhao K., Yang S., Chen S., Li C., Han X., Li J., Hu K., Liu S., Ma M. (2024). Insights into antibiotic and heavy metal resistance interactions in *Escherichia coli* isolated from livestock manure and fertilized soil. J. Environ. Manag..

[15] Majowicz S.E., Musto J., Scallan E., Angulo F.J., Kirk M., O’Brien S.J., Jones T.F., Fazil A., Hoekstra R.M., International Collaboration on Enteric Disease “Burden of Illness” Studies (2010). The global burden of nontyphoidal Salmonella gastroenteritis. Clin. Infect. Dis..

[16] Han J., Aljahdali N., Zhao S., Tang H., Harbottle H., Hoffmann M., Frye J.G., Foley S.L. (2024). Infection biology of *Salmonella enterica*. EcoSal Plus.

[17] Salam M.A., Al-Amin M.Y., Salam M.T., Pawar J.S., Akhter N., Rabaan A.A., Alqumber M.A.A. (2023). Antimicrobial Resistance: A Growing Serious Threat for Global Public Health. Healthcare.

[18] Centers for Disease Control and Prevention (2019). 2019 Antibiotic Resistance Threats Report.

[19] Robertson J., Schonfeld J., Bessonov K., Bastedo P., Nash J.H.E. (2023). A global survey of Salmonella plasmids and their associations with antimicrobial resistance. Microb. Genom..

[20] Carattoli A. (2003). Plasmid-mediated antimicrobial resistance in *Salmonella enterica*. Curr. Issues Mol. Biol..

[21] Hsu P.C., Wang Y.W., Chen B.H., Hong Y.P., Teng R.H., Liu P.Y., Chiou C.S. (2023). Carbapenem resistance in extensively drug-resistant *Salmonella enterica* serovar Agona and AmpC beta-lactamase-producing *S.* Infantis. Microbiol. Spectr..

[22] Gillieatt B.F., Coleman N.V. (2024). Unravelling the mechanisms of antibiotic and heavy metal resistance co-selection in environmental bacteria. FEMS Microbiol. Rev..

[23] Feldgarden M., Brover V., Gonzalez-Escalona N., Frye J.G., Haendiges J., Haft D.H., Hoffmann M., Pettengill J.B., Prasad A.B., Tillman G.E. (2021). AMRFinderPlus and the Reference Gene Catalog facilitate examination of the genomic links among antimicrobial resistance, stress response, and virulence. Sci. Rep..

[24] Galata V., Fehlmann T., Backes C., Keller A. (2019). PLSDB: A resource of complete bacterial plasmids. Nucleic Acids Res..

[25] McKinney W. (2011). Pandas: A Foundational Python Library for Data Analysis and Statistics. Python High. Perform. Sci. Comput..

[26] Jones E., Oliphant T., Peterson P. SciPy: Open Source Scientific Tools for Python. Proceedings of the 9th Python in Science Conference (SciPy 2010).

[27] Mourao J., Marcal S., Ramos P., Campos J., Machado J., Peixe L., Novais C., Antunes P. (2016). Tolerance to multiple metal stressors in emerging non-typhoidal MDR Salmonella serotypes: A relevant role for copper in anaerobic conditions. J. Antimicrob. Chemother..

[28] Williams J.R., Morgan A.G., Rouch D.A., Brown N.L., Lee B.T. (1993). Copper-resistant enteric bacteria from United Kingdom and Australian piggeries. Appl. Environ. Microbiol..

[29] Staehlin B.M., Gibbons J.G., Rokas A., O’Halloran T.V., Slot J.C. (2016). Evolution of a Heavy Metal Homeostasis/Resistance Island Reflects Increasing Copper Stress in Enterobacteria. Genome Biol. Evol..

[30] Hikal A.F., Hasan S., Gudeta D., Zhao S., Foley S., Khan A.A. (2024). The acquired pco gene cluster in *Salmonella enterica* mediates resistance to copper. Front. Microbiol..

[31] Hernandez-Montes G., Arguello J.M., Valderrama B. (2012). Evolution and diversity of periplasmic proteins involved in copper homeostasis in gamma proteobacteria. BMC Microbiol..

[32] Li P., Nayeri N., Gorecki K., Becares E.R., Wang K., Mahato D.R., Andersson M., Abeyrathna S.S., Lindkvist-Petersson K., Meloni G. (2022). PcoB is a defense outer membrane protein that facilitates cellular uptake of copper. Protein Sci..

[33] Li M., Wang K., Tang A., Tang A., Chen A., Huang Z. (2021). Investigation of the Genes Involved in the Outbreaks of *Escherichia coli* and Salmonella spp. in the United States. Antibiotics.

[34] Nascimento A.M., Chartone-Souza E. (2003). Operon mer: Bacterial resistance to mercury and potential for bioremediation of contaminated environments. Genet. Mol. Res..

[35] Naguib M.M., El-Gendy A.O., Khairalla A.S. (2018). Microbial Diversity of Operon Genes and Their Potential Rules in Mercury Bioremediation and Resistance. Open Biotechnol. J..

[36] Boyd E.S., Barkay T. (2012). The mercury resistance operon: From an origin in a geothermal environment to an efficient detoxification machine. Front. Microbiol..

[37] Hui C.Y., Liu M.Q., Guo Y. (2024). Synthetic bacteria designed using ars operons: A promising solution for arsenic biosensing and bioremediation. World J. Microbiol. Biotechnol..

[38] Souza S.S.R., Turcotte M.R., Li J., Zhang X., Wolfe K.L., Gao F., Benton C.S., Andam C.P. (2022). Population analysis of heavy metal and biocide resistance genes in *Salmonella enterica* from human clinical cases in New Hampshire, United States. Front. Microbiol..

[39] Yang H.C., Rosen B.P. (2016). New mechanisms of bacterial arsenic resistance. Biomed. J..

[40] Foley S.L., Johnson T.J., Ricke S.C., Nayak R., Danzeisen J. (2013). Salmonella pathogenicity and host adaptation in chicken-associated serovars. Microbiol. Mol. Biol. Rev..

[41] Algarni S., Ricke S.C., Foley S.L., Han J. (2022). The dynamics of the antimicrobial resistance mobilome of *Salmonella enterica* and related enteric bacteria. Front. Microbiol..

[42] Algarni S., Han J., Gudeta D.D., Khajanchi B.K., Ricke S.C., Kwon Y.M., Rhoads D.D., Foley S. (2023). In silico analyses of diversity and dissemination of antimicrobial resistance genes and mobile genetics elements, for plasmids of enteric pathogens. Front. Microbiol..

[43] Han J., Lynne A.M., E David D., Tang H., Xu J., Nayak R., Kaldhone P., Logue C.M., Foley S.L. (2012). DNA sequence analysis of plasmids from multidrug resistant *Salmonella enterica* serotype Heidelberg isolates. PLoS ONE.

[44] Zhang D., Li S., Zhang X., Zheng S., Zhou D., Hou Q., Li G., Han H. (2025). Epidemiological and biological characteristics of IncR plasmids as multihost antibiotic resistance carriers. Front. Microbiol..

[45] Han J., Pendleton S.J., Deck J., Singh R., Gilbert J., Johnson T.J., Sanad Y.M., Nayak R., Foley S.L. (2018). Impact of co-carriage of IncA/C plasmids with additional plasmids on the transfer of antimicrobial resistance in *Salmonella enterica* isolates. Int. J. Food Microbiol..

